# Unique and Conserved MicroRNAs in Wheat Chromosome 5D Revealed by Next-Generation Sequencing

**DOI:** 10.1371/journal.pone.0069801

**Published:** 2013-07-23

**Authors:** Kuaybe Yucebilgili Kurtoglu, Melda Kantar, Stuart J. Lucas, Hikmet Budak

**Affiliations:** 1 Faculty of Engineering and Natural Sciences, Sabanci University, Orhanlı, Tuzla, Istanbul, Turkey; 2 Sabanci University Nanotechnology Research and Application Center (SUNUM), Sabanci University, Tuzla, Istanbul, Turkey; National Institutes of Health, United States of America

## Abstract

MicroRNAs are a class of short, non-coding, single-stranded RNAs that act as post-transcriptional regulators in gene expression. miRNA analysis of *Triticum aestivum* chromosome 5D was performed on 454 GS FLX Titanium sequences of flow-sorted chromosome 5D with a total of 3,208,630 good quality reads representing 1.34x and 1.61x coverage of the short (5DS) and long (5DL) arms of the chromosome respectively. *In silico* and structural analyses revealed a total of 55 miRNAs; 48 and 42 miRNAs were found to be present on 5DL and 5DS respectively, of which 35 were common to both chromosome arms, while 13 miRNAs were specific to 5DL and 7 miRNAs were specific to 5DS. In total, 14 of the predicted miRNAs were identified in wheat for the first time. Representation (the copy number of each miRNA) was also found to be higher in 5DL (1,949) compared to 5DS (1,191). Targets were predicted for each miRNA, while expression analysis gave evidence of expression for 6 out of 55 miRNAs. Occurrences of the same miRNAs were also found in *Brachypodium distachyon* and *Oryza sativa* genome sequences to identify syntenic miRNA coding sequences. Based on this analysis, two other miRNAs: miR1133 and miR167 were detected in *B. distachyon* syntenic region of wheat 5DS. Five of the predicted miRNA coding regions (miR6220, miR5070, miR169, miR5085, miR2118) were experimentally verified to be located to the 5D chromosome and three of them : miR2118, miR169 and miR5085, were shown to be 5D specific. Furthermore miR2118 was shown to be expressed in Chinese Spring adult leaves. miRNA genes identified in this study will expand our understanding of gene regulation in bread wheat.

## Introduction

MicroRNAs (miRNAs), a class of short (∼21 nt) non-coding, single stranded RNAs, are highly conserved across plant species. Plant miRNAs have been shown to play a critical role in diverse biological processes including growth, development, adaptation to biotic and abiotic stresses, signal transduction and protein degradation as well as their own biogenesis [1]–[13]. They act as post-transcriptional regulators in gene expression via target specific cleavage and translational repression [14]–[16]. Mature miRNAs are processed from long primary transcripts (pri-miRNAs) in a multistep manner. Pri-miRNAs that are generated from miRNA genes in the nucleus are then cleaved by Dicer-like1 nuclease (DCL1) to produce a precursor (pre-miRNA) that folds into a hairpin structure. This hairpin is further cleaved to excise a double stranded miRNA/miRNA* fragment from the stem of the hairpin [17], [18]. The duplex is then methylated by HEN1 and exported to the cytoplasm by a protein called HASTY, an exportin-5 homologue [19]. Shortly after this, the single-stranded miRNA or miRNA* is incorporated into the RNA-induced silencing complex (RISC). In turn the RISC complex regulates specific target mRNAs, usually by cleavage at the miRNA complementary sequence [16], [18], [20], [21].

Since the first plant miRNA was discovered in *Arabidopsis* [22], more than 3000 plant miRNAs have been found either by direct cloning of small RNA libraries or bioinformatic prediction based on sequence and secondary structure conservation [23]. To date, 3228 plant miRNAs have been identified in various plants and submitted to miRBase (release 19.0, August 2012).

Due to the high abundance of small interfering RNAs (siRNAs), which comprise the majority of the plant small RNA pool and resemble miRNAs in length and sequence-specific function, miRNA identification in plants is complicated. The major difference between miRNAs and siRNAs is the processing of their precursors; miRNAs are derived from imperfectly paired single-stranded stem-loop structures [23], [24], whereas siRNAs are derived from long, perfectly paired double-stranded RNAs [25], [26]. They also differ in mode of action; miRNAs function at the post-transcriptional level through mRNA degradation or transcriptional repression, while siRNAs trigger DNA methylation, histone modification and mRNA degradation at transcriptional and post-transcriptional levels [7], [27], [28]. In order to distinguish miRNAs from other RNAs and confidently annotate miRNAs, stringent criteria have been specified [29]. miRNAs are highly conserved between species. Thus sequence and secondary structure homology have been utilized to predict the novel miRNAs conserved in other organisms by computational analysis. This approach is also useful in detecting miRNAs expressed at very low levels. Predicted miRNAs ultimately have to be verified experimentally to be confirmed as ‘miRNA’ [29].

Furthermore, the bread wheat genome (∼17 Gb) is known to contain highly repetitive sequences [30]. Recent studies have shown that repeat elements, especially those transposable elements (TEs) containing inverted repeats that could fold into hairpin-like structures, have contributed to miRNA biogenesis [31], [32].Co-localization of TEs with miRNAs was initially studied in *Arabidopsis thaliana (A. thaliana)* and *Oryza sativa (O. sativa),* and it is proposed that some miRNA genes have been derived from DNA transposons,frequently the miniature-inverted TEs (MITEs) [33]. Additionally Li and colleagues found a number of miRNAs homologous to TEs in plant species including bread wheat, supporting the idea of domestication of TEs into miRNA genes [34]. Some of the plant miRNAs deposited in miRBase were also found to be TE derived [33].

In this study we utilize next-generation sequencing data of flow-sorted individual chromosome arms for computational identification of miRNAs located on wheat chromosome 5D. Improvements in chromosome sorting techniques have facilitated genomic studies of the polyploid wheat genome by reducing the template to a manageable size [35]. By this approach, putative miRNAs have been identified at the subgenomic level, and these miRNAs were mined for the purpose of understanding their roles in the regulation of growth, development and biological processes.

## Results

### Identification and characterization of conserved miRNAs in long and short arms of wheat chromosome 5D

A total of 5,940 known plant mature miRNA sequences derived from 67 plant species were obtained from miRBase. After elimination of duplicate mature miRNA sequences, 3,228 mature miRNAs were used as query in BLASTn searches against 937,264 454 GS FLX sequence reads (1.34x coverage) for the short arm and 2,271,366 reads (1.61x coverage) forthe long arm respectively, corresponding to a total of 3,208,630 *T. aestivum* chromosome 5D sequences.

**Figure 1:**
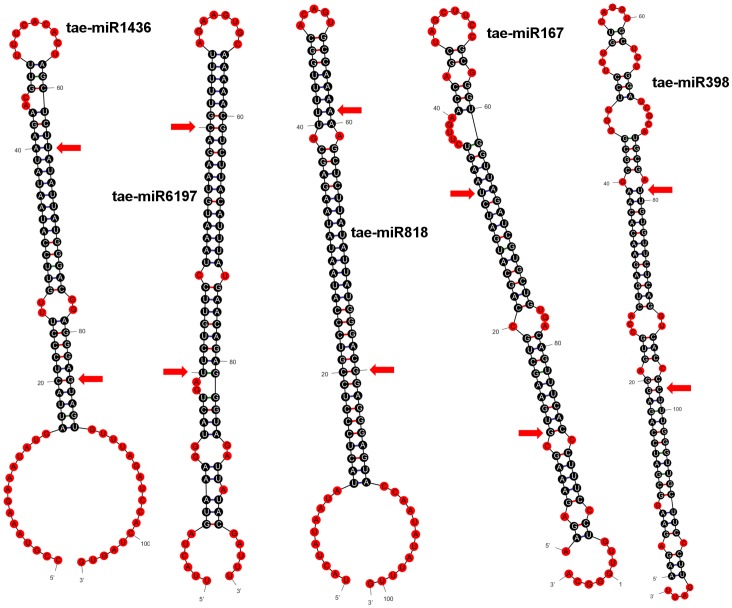
Identified pre-miRNA stem-loop structures of selected miRNAs on chromosome 5D. Mature miRNA sequence start and end points are designated with arrows. Structures are predicted using UNAFold (an implementation of Zuker algorithm).

After using UNAfold,an implementation of the Zuker folding algorithm, 55 different miRNAs were identified from their predicted pre-miRNA stem-loop structures. Of these,13 miRNAs were found to be specifically present in the long arm, whereas 7 were specific to the short arm of 5D. (Figure 1, Data S1: Table 1).

**Table 1:** miRBASE deposited targets for homologs of 5D *T.aestivum* miRNAs.

miRNA Name	Species of target was identified	Experimentally conformed target
miR167	ath/osa	Auxin response factors
miR395	ath/osa	ATP sulphurylase
miR160	ath/osa	Auxin response factors

ath-Arabidopsis thaliana; osa-Oryza sativa.

Allowing for up to 3 mismatches from a known plant mature miRNA, 654 and 428 potential mature miRNA sequences were identified in 5DL and 5DS respectively; overall, the 55 5D miRNAs contained 926 potential mature miRNA sequences. Corresponding stem-loop structures for each new miRNA sequence that passed the 3 mismatch criteria were analyzed for miRNA characteristics.

Average sequence length for identified pre-miRNAs (130.022±39.39 nt, with a median of 122 nt) and mature miRNAs (20.9±1–36 and a median of 20), and average %GC content for pre-miRNAs (40.45% ±8.60 with a median of 38.022%, and minimum and maximum values of 26.06% and 67.01%) were calculated. Minimal folding free-energy index(MFEI), which is calculated from the minimal folding-free energy (MFE), sequence length and %GC content of the pre-miRNA, differentiates miRNAs with typically higher MFEIs (>0.67) from other types of cellular ssRNAs for which MFEIs were previously characterized; transfer RNAs (0.64), ribosomal RNAs (0.59), and mRNAs (0.62–0.66) [36]. The descriptive statistics for predicted wheat pre-miRNA MFEI values were; average:1.19±0.27, median: 1.19±0.27, minimum: 0.68, and maximum: 2.07. The low negative MFE values show higher stability of the predicted miRNA. The MFEs of the miRNAs we identified had an average of −61.82±23.44 and median of −59.7 kcal/mol; a minimum of −161.5 and a maximum of −23.4, which correlates well with previous plant miRNA identification studies [10], [11], [37]. Average MFEI values of 5DL and 5DS miRNAs were 1.19±0.27 with a median of 1.16 and 1.19±0.27 with a median of 1.19 respectively (Data S2: Table 2 and 3).

**Table 2:** Potential target genes and their predicted functions for 14 newly identified miRNAs in wheat chromosome 5D.

miRNA	Target Accession (DFCI Index)	Targeted protein	Possible function
miR3700	TC371315, TC371563,TC372626, TC437840	Polyubiquitin	
	TC412324	P-type H+-ATPase	hydrolase activity
	TC409264	SET domain protein-like	transferase activity
miR482	TC425646, TC421071	NBS-LRR type RGA	ion binding activity
	CA678894, TC393142, TC421789, TC410768	OJ000223_09.9 protein	unknown
	TC402759	Ribosomal L14 protein	ribosome
	CO348589	Short-chain alcohol dehydrogenase	oxydoreductase activity
miR5068	CA605881	Acidic ribosomal protein	structural constituent of ribosome
	BE217043	Phenylalanine ammonia-lyase	lyase activity
	CV066196	Allergen C-C	extracellular region
miR5161	CA647968	LEM3-like	phospholipid transport
	CJ530860	SNF7 protein-like	protein transport
	CK204669	6-phosphogluconolactonase	hydrolase activity
	TC387475	Pyruvate kinase	transferase activity
	TC414519	Acyl carrier protein 3, chloroplast precursor	cofactor binding activity
	TC414519	Acyl carrier protein 3, chloroplast precursor	lipid metabolic activity
	TC379110	Calnexin	metal ion binding activity
	CV066415	Aspartic proteinase	hydrolase activity
miR5205	TC412474	60S ribosomal protein L44	structural constituent of ribosome
	TC412475	60S ribosomal protein L45	ribosome
	TC444618	Abscisic stress ripening-like protein	response to stress
	TC430550	Aldehyde oxidase-2	oxydoreductase
	TC420918	Arf6/ArfB-family small GTPase	nucleotide binding
	TC377933	ATP dependent Clp protease ATP-binding subunit ClpX1	hydrolase
	TC377934	ATP dependent Clp protease ATP-binding subunit ClpX2	ion binding
	AL821953	CHY zinc finger family protein, expressed	metal ion binding
	CJ883403	Cysteine synthase	lyase activity
	TC385385	Exoglucanase precursor	hydrolase
	TC413453	F-box domain containing protein, expressed	NA binding TF activity
	CD880846	Flowering promoting factor-like 1	response to hormone
	TC402657	Glyceraldehyde-3-phosphate dehydrogenase, cytosolic	nucleotide binding
	TC402658	Glyceraldehyde-3-phosphate dehydrogenase, cytosolic	oxydoreductase
	TC384047	Glycosyltransferase	transferase activity
	TC452182	Glyoxalase II	catalytic activity
	CK214985, CK211600,CK216047, CK214076	High light protein	responce to stimulus
	CJ563368	Lipid transfer protein-like	lipid transport
	TC389043	Malate dehydrogenase [NADP], chloroplast precursor	oxydoreductase
	DR732905	MIKC-type MADS-box transcription factor WM22A	NA binding TF activity
	CN012529	Mitochondrial transcription termination factor-like protein	
	TC407053	NADPH-cytochrome P450 reductase	oxydoreductase
	TC398674	Phytoene dehydrogenase, chloroplast/chromoplast precursor	oxydoreductase
	CD879785	Probable protein ABIL1	cytoskeleton
	TC433753	Proline-rich spliceosome-associated protein-like	metal ion binding
	TC459951	Protein kinase domain containing protein, expressed	transferase activity
	TC406931	Ribosomal protein L7	ribosome
	TC449066,TC390499	S-adenosylmethionine decarboxylase proenzyme	lyase activity
	CD879878	Serine-threonine protein kinase	transferase activity
	TC395950	STF-1	transferase activity
	TC453849	U2AF large subunit	nucleotide binding
	TC377545	Ubiquitin-conjugating enzyme-like protein	ligase activity
	TC383983	Utp14 protein, expressed	macromolecular component
miR5281	AL821953	CHY zinc finger family protein, expressed	metal ion binding
	TC398674	Phytoene dehydrogenase, chloroplast/chromoplast precursor	oxydoreductase
	TC389043	Malate dehydrogenase [NADP], chloroplast precursor	oxydoreductase
	BQ166729	HAT family dimerisation domain containing protein, expressed	NA binding TF activity
	TC383983	Utp14 protein, expressed	macromolecular component
	TC415094	Cell division inhibitor-like	nucleotide binding
	TC424763	Protein kinase domain containing protein, expressed	transferase activity
	TC425550	Lipase class 3-like	hydrolase
	TC430886	SMC5 protein	chromosomal part
miR5387	TC437702	Histone H3.2	protein binding
	BE637541	LEA protein	drought stress responsive
	TC387640	Heterogeneous nuclear ribonucleoprotein A2/B1-like	nucleotide binding
miR5568	TC397912	Adenosine diphosphate glucose pyrophosphatase precursor	nuclear reservoir activity
	CJ936328	Alpha-L-arabinofuranosidase/beta-D-xylosidase isoenzyme ARA-I	hydrolase
	TC420918	Arf6/ArfB-family small GTPase	ion binding
	CJ883403	Cysteine synthase	lyase activity
	TC401164	Fb14	mitochondira
	TC446402	Glutathione gamma-glutamylcysteinyltransferase 1	transferase activity
	TC452182	Glyoxalase II	catalytic activity
	CK214076, CK214985, CK216047, CK211600	High light protein	responce to stimulus
	TC389043	Malate dehydrogenase [NADP], chloroplast precursor	oxydoreductase
	CN012529	Mitochondrial transcription termination factor-like protein	
	TC398674	Phytoene dehydrogenase, chloroplast/chromoplast precursor	oxydoreductase
	CD879785	Probable protein ABIL1	cytoskeleton
	TC392962	Serine protease-like protein	hydrolase
	TC395950	STF-1	transferase activity
	TC383983	Utp14 protein, expressed	macromolecular component
	TC393805	Protein kinase	transferase activity
	TC390967	Glutaredoxin-C1 precursor	electron carrier activity
	TC390968	Glutaredoxin-C1 precursor	oxidoreductase
	DR732905	MIKC-type MADS-box transcription factor WM22A	NA binding TF activity
	AL821953	CHY zinc finger family protein, expressed	metal ion binding
	TC405376	Serine carboxypeptidase family protein, expressed	hydrolase
	CK211600, CK216047, CK214985, CK214076	High light protein	
	TC392962	Serine protease-like protein	hydrolase
	TC379065	Proteinase inhibitor	enzyme regulatory activity
	TC452003	Subtilisin-chymotrypsin inhibitor 2	enzyme regulatory activity
miR6191	CJ868604	Transcriptional activator-like	
	TC371963	Alpha-1,2-fucosidase	hydrolase
	TC453681	Ribosomal protein L15	structural constituent of ribosome
	TC400735	SET domain containing protein, expressed	
miR6197	TC397912	Adenosine diphosphate glucose pyrophosphatase precursor	metal ion binding
	AL821953	CHY zinc finger family protein, expressed	metal ion binding
	TC379438	Germin-like protein 1 precursor	metal ion binding
	TC398842	Glutamine synthetase	ligase activity
	TC446402	Glutathione gamma-glutamylcysteinyltransferase 1	
	TC452182	Glyoxalase II	
	TC380096	haloacid dehalogenase-like hydrolase family protein	transferase activity
	TC438131	Ice recrystallisation inhibition protein	frost resistance?
	TC415220	Kinase, CMGC CDKL	transferase activity
	TC391197	Leucine Rich Repeat family protein, expressed	ion binding
	TC369729	LRK14	transferase activity
	CA601255	Membrane bound O-acyl transferase MBOAT	transferase activity
	CK200742	Metallo-beta-lactamase-like	hydrolase activity
	CJ861664	Phosphatidylinositol transfer-like	transport activity
	TC442517	Protein HVA22	respond to stress
	TC440668	Ribosomal Pr 117	structural constituent of ribosome
	CJ868604	Transcriptional activator-like	
	TC383983	Utp14 protein, expressed	
	TC419868	Zinc finger protein	metal ion binding
miR6219	CO348282	Mono-or diacylglycerol acyltransferase	transferase activity
	CA697004	Auxin-repressed protein-like protein ARP1	
	TC376658	Peroxidase 8	oxidoreductase
	CA617623	Vacuolar ATP synthase 16 kDa proteolipid subunit	transporter activity
	TC378198	Cinnamyl alcohol dehydrogenase	oxidoreductase
	CA731724	Glycosyltransferase	transferase activity
miR6220	CK211600, CK216047,CK214985, CK214076	High light protein	responce to stimulus
	TC377933	ATP dependent Clp protease ATP-binding subunit ClpX1	prt binding TF activity
	TC433753	Proline-rich spliceosome-associated protein-like	
	TC392962	Serine protease-like protein	hydrolase
	TC453352	Probable esterase PIR7A	metal ion binding
miR6224	TC402657	Glyceraldehyde-3-phosphate dehydrogenase, cytosolic	nucleotide binding
miR950	TC400027	C2H2 zinc-finger protein	metal ion binding
	TC441942	60S ribosomal protein L19-like protein	structural constituent of ribosome

**Table 3:** List of ESTs deposited in GenBank that have high homology to predicted 5D miRNAs.

miRNA Name	EST (Genbank)
miR1122	CJ632148.1
miR1439	CJ510559.1
miR1436	AL816538.1
miR167	CJ846906.1, CJ833771.1
miR5205	CJ631979.1, CJ523432.1
miR1136	CJ665546.1, CD876589.1, BE591362.1

miRNA families were taken to match ESTs if they had hits above the following thresholds: query coverage > = 99%, maximum identity > = 98%.

### miRNA representation analysis

According to the results 5DL showed higher variety and representation of miRNAs than 5DS, as might be expected from its larger size. Twelve and 5 miRNAs were represented by only one putative pre-miRNA in the 5DS and 5DL arms, respectively. Eleven miRNAs were only detected at a single locus throughout the whole chromosome. The absolute copy number of each miRNA cannot be determined with certainty as some genomic miRNAs may be covered by more than one sequence read, while others may not be covered at all; however, the representation of each miRNA within the dataset provides a useful estimate of its prevalence on the chromosome. 5D miRNAs with the highest apparent representation (over 100 copies) were miR1117, miR1120, miR1139, miR1436, miR5049 in 5DS; miR1117, miR1120, miR1122, miR1131, miR1135, miR1136, miR1436, miR5049 in 5DL (Data S3; Table 1). The amount of 5D miRNA representation differed widely between miRNAs, and was found to be as high as 117 and 206 copies of a single putative miRNA present in 5DS and 5DL respectively.

### Potential miRNA targets

Predicted 5D miRNAs were searched manually in miRBase to identify those with confirmed target mRNAs in other plant species. Targets were found for 3 miRNAs; for one miRNA unique to 5DL, and two miRNAs unique to 5DS; none of miRNAs with known targets were identified in sequence reads from both arms (Table 1). As a further analysis, using psRNATarget software, possible targets were retrieved for one predicted *T. aestivum* mature miRNA sequence corresponding to each identified miRNA family. In this analysis, possible targets were predicted for a total of 55 miRNA sequences, 48 (out of 48) from 5DL, 40 (out of 42) from 5DS and 33 (out of 35) miRNAs located in both chromosome arms (Data S4: Table 1,2). Putative wheat miRNA target genes varied in sequence and function, and most of them were classified as transcription factors, functional proteins in plant metabolism, and protein subunits. Potential targets of the newly identified miRNAs were listed in Table 2.

### Elimination of known repeat sequences encoding miRNAs

The high representation of some of the putative miRNAs detected on chromosome 5D suggests that some or all of their apparent loci could be repetitive sequences. Therefore, all putative pre-miRNA hairpin sequences detected above were compared with a database of known wheat repetitive elements (see Materials and Methods). As a result, 83.84% and 84.38% of the 5DL and 5DS sequences were masked as repeats. Both Class I and Class II TEs were present in potential miRNA sequences with Class II DNA transposons being the predominant repeat elements; 81.54% and 81.98% of putative miRNA sequences were classified as DNA transposon elements in 5DL and 5DS, respectively (Data S5: Table 2 and 3). The composition of the repeats present in both chromosome arms was very similar and mostly consisted of MITEs from the Mariner family, followed by CACTA elements (Data S5: Table 1). Interestingly,the 5DL chromosome arm sequences were masked slightly less than 5DS chromosome arm sequences. The distribution of repeat elements also showed slight differences between 5DL and 5DS; differences in composition and distribution of TEs between different chromosomes, and even different regions of the same chromosome in wheat species have been reported previously [38]–[40].

### Evidence for wheat chromosome 5D miRNA expression

Unlike siRNAs, miRNAs are generated from pri-miRNA transcripts, which are capped and polyadenylated in the same manner as protein-coding mRNAs [41]. Therefore, pri-miRNA sequences may be found in EST databases, albeit rarely [42]. As of January 2013, 1,286,372 *T.aestivum* ESTs had been submitted to the NCBI database (http://www.ncbi.nlm.nih.gov/dbEST/), making this a useful resource for attempting to confirm expression of putative wheat miRNAs. For each new miRNA detected in long and short chromosome arms, one corresponding pre-miRNA sequence was searched against the expressed sequence tag (EST) databases of *T. aestivum* using NCBI BLASTn. Hits above a threshold query coverage of 99% and maximum identity of 98%were recorded for each potential miRNA. To identify candidate pre-miRNA coding ESTs, all EST matches were compared to the non-redundant protein database at NCBI using blastx. All ESTs matching any protein sequence at an e-value of 1e^−03^ or lower were considered to be protein-coding, and were eliminated. A total of 6 (out of 55) miRNAs; 4 (miR1136, miR1436, miR167, miR5205) from 5DL, 4 (miR1122, miR1136, miR1436, miR1439) from 5DS, and 2(miR1136 and miR1436) of which were located in both chromosome arms matched an EST with no significant similarity to known proteins (Table 3), suggesting that these putative pre-miRNA sequences are transcribed. The remaining putative pre-miRNAs may also be transcribed, but absent from the available EST databases.

### Identification of chromosome specific expression of miRNAs in *O.sativa* and *B.distachyon*

To find out whether miRNA coding sequences identified in wheat chromosome 5D are conserved in other grass species, the 5D miRNA sequences were used to search thecomplete genome sequences of *B.distachyon* and *O.sativa,* using the sameprocedure described for identification of conserved miRNAs in chromosome 5D, except that 100% identity of the mature miRNA sequence was required. miRNAs that were found to be specifically present in one or more chromosomes were recorded. In our ongoing analysis of the same dataset used in this study, syntenic regions of *B.distachyon* and *O.sativa* for both 5DL and 5DS chromosome arms have been delineated (Lucas *et al*., unpublished). According to these results, chromosome arm 5DL has regions syntenic to chromosomes Bd1& Bd4, and *O.sativa*chromosomes 3& 9, whereas chromosome arm 5DS was found to have syntenic regions to chromosome Bd4 and *O.sativa* 12. Based on this analysis, miR1133 and miR167 were found to be present not only on 5DS but also in the syntenic region of Bd4 (Table 4). None of the 5DL specific miRNAs gave hits to the corresponding syntenic regions of *B.distachyon* and *O.sativa* chromosomes.

**Table 4:** 5D short arm miRNAs that gave hits to *B.distachyon.*

	Chr1	Chr2	Chr3	Chr4	Chr5
miR1127		*			
miR1128	*	*	*	*	*
**miR1133**				*	
miR1135					*
miR1139		*	*	*	
miR1439	*	*	*	*	*
**miR167**				*	
miR395	*				*
miR5049	*	*	*	*	*
miR5175	*	*			*
miR5180	*		*		*
miR5203	*	*	*		*

Bold miRNAs gave the best results that they were syntenic to Bd4.

### Experimental Evidence for localization of predicted pre-miRNA coding regions on 5D chromosome

**Figure 2:**
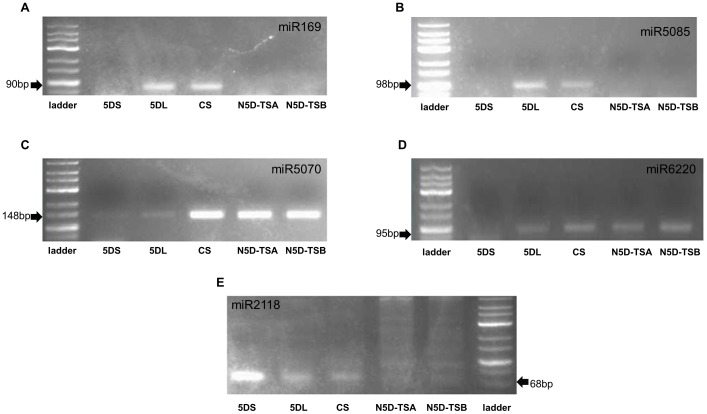
Pre-miRNA coding regions on long and short arms of the 5D chromosome. PCR screening of pre-miRNA coding sequences in flow sorted 5D short and long chromosome arms (5DS and 5DL); *Triticum aestivum* L. cv Chinese Spring (CS) and nullitetrasomic lines (N5D-T5A and N5D-T5B) (A) pre-miR169 (B) pre-miR5085 (C) pre miR5070 (D) pre-miR6220 (E) pre-miR2118.

**Figure 3:**
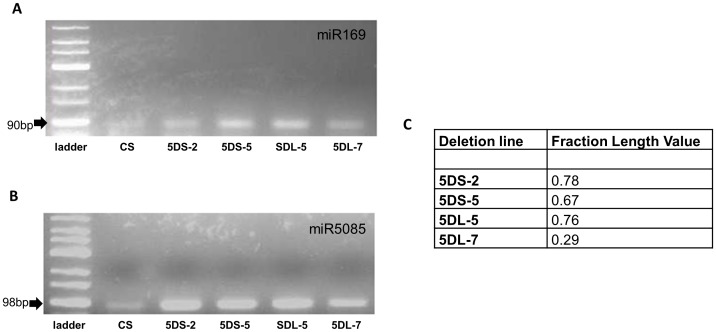
Screening of pre-miRNA coding regions specific to 5DL with wheat group-5 deletion series. PCR screening of 5DL specific pre-miRNA coding sequences (A) pre-miR169 (B) pre-miR5085 in *Triticum aestivum* L. cv Chinese Spring (CS) deletion lines (5DS-2, 5DS-5, 5DL-5, 5DL7) (C) Fraction length values of deletion lines.

**Figure 4:**
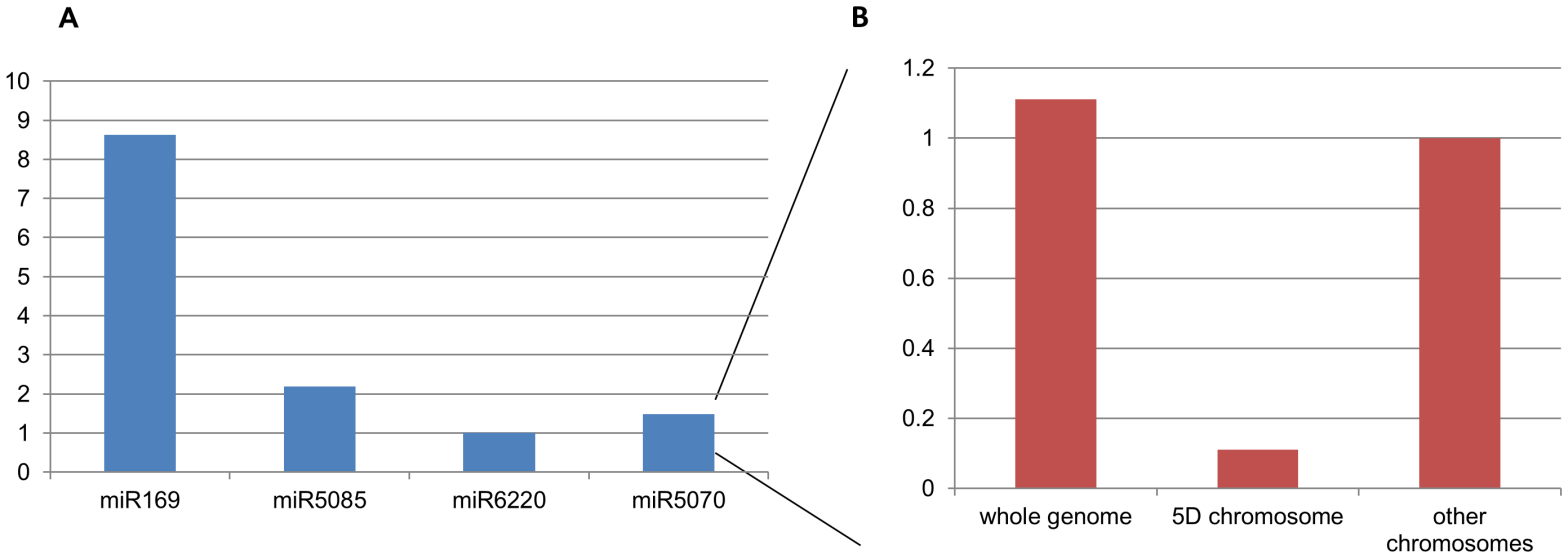
Quantification of miRNA gene copy number. q-RT PCR (A) Five miRNA coding regions (pre-miR169, pre-miR5085, pre-miR6220 and pre-miR5070) in *Triticum aestivum* L. cv Chinese Spring (CS) B) Levels of non 5D-specific miR5070, detected by qRT-PCR in nullitetrasomic line (N5D-T5A) and *Triticum aestivum* L. cv Chinese Spring (CS).

In order to verify 5D chromosome localization, five of the predicted pre-miRNA coding regions,were amplified from flow sorted 5D chromosome arms by PCR. screening. Our experimental results supported our *in silico* predictions: 5DS was verified to harbour regions coding for pre-miR2118 and pre-miR5070, and 5DL was confirmeded to contain both of the above plus pre-miR6220, pre-miR5085 and miR169 coding regions. Furthermore, in order to confirm that these pre-miRNAs are specifically located on chromosome 5D, we also screened gDNA from CS and nullitetrasomic lines. pre-miR169, pre-miR5085 and pre-miR2118 coding regions were found to be 5D chromosome-specific. miR2118 was shown to be located on both arms of the 5D chromosome (5D specific), while miR5085 and miR169 were found to be specific to the long arm (Figure 2). Furthermore, to map the chromosomal positions of 5DL specific miRNAs, gDNA of group-5 deletion lines were also screened. Coding regions of both 5DL specific pre-miRNAs (pre-miR5085, pre-miR169) were found to be located between the breakpoint of 5DL-7 (FL : 0.29) and the centromere (Figure 3). Quantification with real-time PCR using CS gDNA suggested that coding regions of the selected pre-miRNAs had variable copy number: pre-miR169, pre-miR5085 and pre-miR5070 were shown to have approximately 8.6, 2.2 and 1.5 fold more copies than pre-miR6220. Gene copy number of pre-miR6220 was also separately evaluated with qRT-PCR in nullitetrasomic lines in comparison to CS, to determine its gene copy number restricted to the 5D chromosome. Approximately 9% of pre-miR6220 coding sequence copies from the whole wheat genome were observed to be located on chromosome 5D (Figure 4).

### Experimental evidence for expression of pre-miR2118 from wheat 5D chromosome

**Figure 5:**
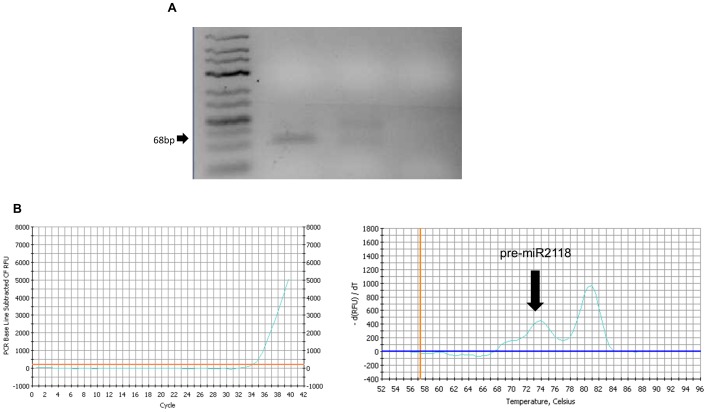
Evidence for pre-miR2118 expression in wheat. Expression of pre-miR2118 in *Triticum aestivum* L. cv Chinese Spring (CS) (A) Endpoint PCR results (B) qRT-PCR results.

In order to show expression of selected pre-miRNAs (pre-miR2118, pre-miR169, pre-miR5085, pre-miR6220, pre-miR5070), RT-PCR and qRT-PCR was performed using Chinese Spring cDNA. Expression of pre-miR2118 in adult leaves of wheat, grown under standard greenhouse conditions was shown. Expression was not unequivocally confirmed for the other 5D-specific pre-miRNAs, but as individual miRNA expression is frequently tissue/developmental stage/environmental condition specific, their expression may be detectable under specific conditions that were not tested here, most probably stress conditions (Figure 5).

## Discussion

The advent of next-generation high-throughput sequencing, chromosome sorting techniques and complementary bioinformatics tools have provided better approaches to identify miRNAs systematically at the sub-genomic level. The development of chromosome sorting techniques allows chromosome based sequencing, followed by identification of putative miRNA genes. Being one of the most important cereal crops in the world, understanding wheat genes and their regulation is a high priority; identifying wheat miRNAs and their targets is an important step in characterizing gene expression and regulation at the post-transcriptional level. Small RNA library sequences enable the identification of novel miRNAs which are present under the conditions in which the library RNA was collected [43], [44]; searching chromosomal sequences for miRNAs is a complementary strategy, with the advantage of detecting potential miRNAs present in the genome that are only expressed at low levels, or under conditions not represented by the small RNA libraries.

In this study, flow-sorted wheat chromosome 5D 454 sequence reads from *T. aestivum* L. var. “Chinese Spring” were used, and using in-house Perl scripts (see Materials and Methods) the first identification of conserved miRNAs in this chromosome was performed. To date 3,228 unique plant miRNA sequences have been deposited in miRBase. After a BLAST search based on sequence homology and conservation of pre-miRNA secondary structure, 55 putative conserved miRNAs were identified in5D, in which 13 miRNAs were specifically found to be present on 5DL and 7 on 5DS.The remaining 35 were found in both arms (Data S1: Table 1). Considering the total read count of 937,264 reads and 2,271,366 reads for 5DS and 5DL respectively, together with all analysis, it is notable that the long arm of the chromosome was shown to have a higher variety and representation of miRNAs compared to short arm. This is in accordance with the previous EST mapping studies in which 5DS has mapped roughly half the number of ESTs mapped on 5DL [45]. Bearing in mind the relative size of the chromosome arms, distribution of putative miRNA sequences seems to be consistent across the chromosome [46].

A total of37 miRNA families found in *T.aestivum* have previously been deposited in miRBase [42]–[44], [47]. Of the putative miRNAs reported here, 17 (out of 48) in 5DL and 14 (out of 42) in 5DS, making a total of 18 (out of 55) in the whole chromosome were previously reported to be *T. aestivum* miRNAs in miRBase. The remaining 37 putative miRNAs have not yet been confirmed to be expressed in *T. aestivum.* Conversely, 19 previously reported *T.aestivum* miRNAs were not detected in our dataset, meaning that the coding sequences for these miRNAs are probably not located on chromosome 5D.

Compared to a previous study also carried out by our group, 36 predicted miRNAs (out of 48) in 5DL and 24 (out of 42) in 5DS were also present in wheat chromosome 4A [48] (Data S1: Table 1 and 2).

According to the representation analysis 12 (out of 42), 5 (out of 48), 11 (out of 55) potential miRNAs were represented only once in 5DS, 5DL and the entire chromosome respectively. All of the highly represented miRNAs with over hundred copies were previously identified miRNAs. Eight out of 14 newly identified miRNAs with 10 or fewer copies were classed as “low represented” (Data S3: Table 1). These low represented miRNAs are more likely to be functional miRNA genes, compared to those with higher copy numbers which are probably repeat elements [34]. However, computational miRNAs remains putative until they are experimentally validated. Moreover, miRNA copy number may have a role in the level of regulation of its target; only if that miRNA is highly expressed it is more likely to have a greater effect on target regulation.

Considering the high repetitive content of wheat genome, repeat analysis was performed for the putative miRNAs detected on chromosome 5D. According to the results, Class II DNA Transposons were found to be the predominant repeats found in putative miRNAs from both arms, most frequently MITEs from the Mariner subfamily. CACTA sequences, Harbinger and Mutator sub-families were also detected in masked miRNA sequences. Since MITEs possess mostly palindromic terminal inverted repeat (TIR) sequences that can fold into miRNA-like hairpin structures [14],MITE-derived hairpins could be processed by DCL1, giving rise to mature miRNA sequences [33], [49]. Previously, a number of miRNA genes were found to be derived from TE sequences including osa-miR437 and osa-miR818, both of whichwere also found in *T.aestivum* chromosome 5D[49]–[53]. Due to its repeat rich nature, wheat may have utilized the step-wise model proposed by Piriyapongsa and Jordan [33]to explain how miRNAs could have evolved from TEs,(particularly MITEs) [49], [54].Furthermore, miR5021 corresponds to degenerate trinucleotide repeats and has not been confirmed in any species apart from *A.thaliana*, and so apparent matches to this miRNA are not likely to be true miRNA coding sequences. On the other hand, non-repetitive miRNAs(or non-repeat related miRNAs) all had low representation, with less than 20 hits across the chromosome. However, three of the highly represented miRNAs (miR1122, miR1136, miR1436) with more than 100 copies also gave EST hits (Table 3). In total,6 out of 55 putative miRNAs gave hits to ESTs, again suggesting their expression from wheat chromosome 5D. Expression of the remaining putative pre-miRNAs cannot be ruled out, as the EST database is unlikely to be exhaustive.

According to other research in our lab, chromosome arm 5DL has syntenic regions to chromosomes Bd1& Bd4 and *O.sativa* chromosomes 3 and 9, whereas 5DS was found to have syntenic regions to Bd4 and *O.sativa* chromosome 12 (Lucas *et al.*, unpublished), but most of the miRNAs detected in this study were not syntenically conserved. This indicates that even conserved miRNA sequences have undergone more chromosomal translocations than conserved protein-coding genes since the separation of wheat from *B.distachyon*.

**Figure 6:**
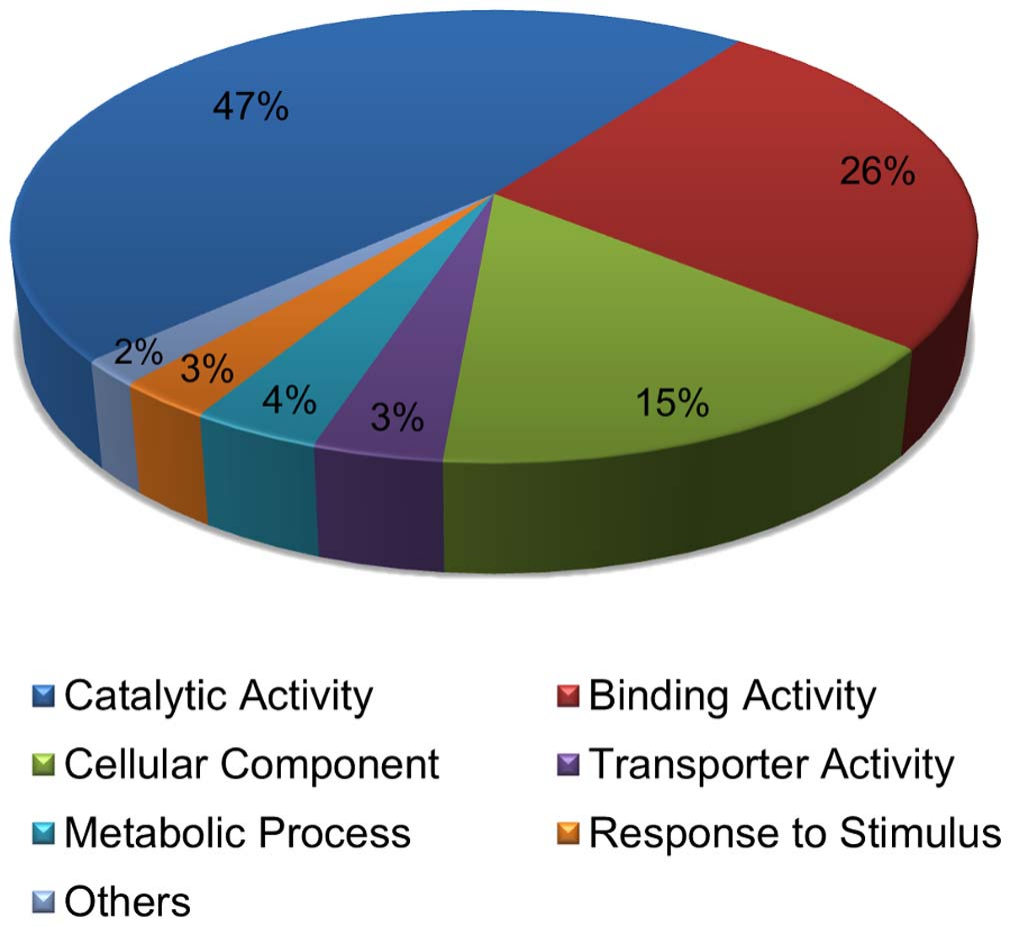
Distribution of possible target functions. Possible functions of newly identified 14 miRNA targets are shown on the pie-chart graph.

Target prediction of miRNAs is widely accepted as an important step towards understanding the role of miRNAs in regulation. All of the putative wheat miRNAs on chromosome 5D were found to have predicted or experimentally confirmed targets, involved in biological or metabolic processes and in stress responses (Data S4: Table 1, 2). The majority of the predicted targets of newly identified miRNAs are involved in a broad range of biological and molecular functions, such as hydrolase activity (miR3700; TC412324), nucleic acid binding transcription factor activity (miR5205; TC413453), transferase activity (miR5568; TC446402, TC395950), oxidoreductase activity (miR482; CO348589), metal ion binding activity (miR6197; AL821953) and response to stresses (miR5387; BE637541) such as drought (Figure 6).

Independent studies in different plant species including *A. thaliana, O. sativa,*and *Populus trichocarpa*showed drought stress responsiveness of miR160,miR167, miR169, miR1125, and miR398, which were also found in wheat chromosome 5D[55]–[57].

In addition to this; other drought related proteins such as late embryogenesis abundant protein (LEA) [58], [59], HVA22 [60], aquaporin [61] and calmodulin-like protein [62] were also found to be targeted by miR5387, miR6197/miR1118, miR1117/miR437 and miR1133 respectively.

Our target analysis show that the majority of the miRNAs have more than one potential regulatory target, conversely one target could be regulated by more than one miRNA. This observation supports the idea that miRNA studies should focus on a regulatory network in which more than one miRNA with different targets are involved (Table 2) [63].

**Figure 7:**
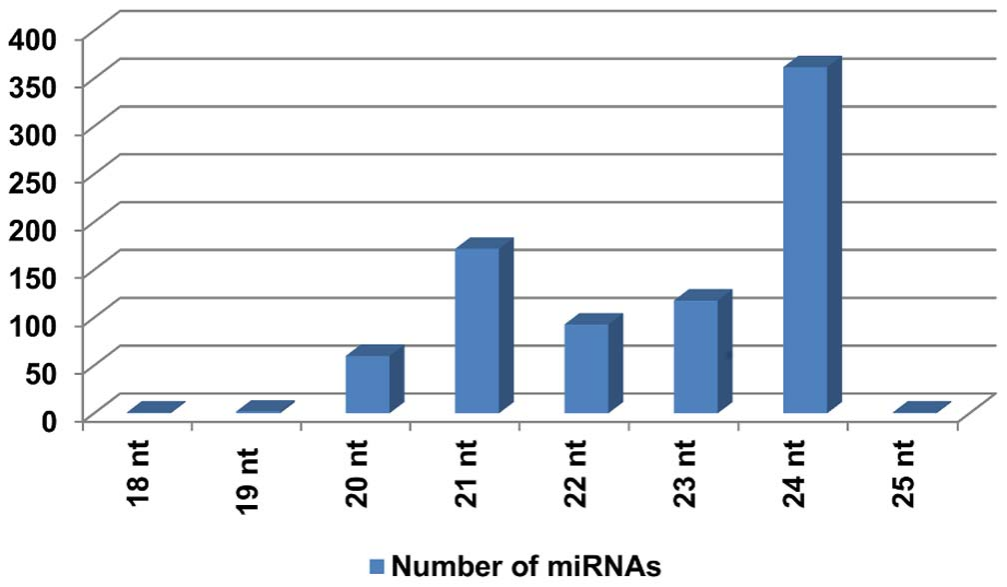
Distribution of different sizes of miRNAs on 5D chromosome. Sizes and numbers of miRNAs on 5D chromosome distribution is shown on the graph.

Size distribution of miRNAs is important to their function. Previous studies have shown that 22 nt miRNAs are more likely to trigger siRNA biogenesis from their target transcripts [64]. Experimental analysis showed that the Argonaute (AGO) proteins have important roles to sort and load mature miRNA duplexes and 22 nt mature miRNA sequences were most effectively sorted and loaded onto the AGO (Figure 7) [65].

In this study, five pre-miRNA (miR169, miR5085, miR2118, miR6220, miR2118) coding sequences were verified to be located to the 5D chromosome (Figure 2). qRT analysis showed that the gene copy numbers of these miRNAs were highly variant (Figure 4). Three of these pre-miRNAs (miR169, miR5085, miR2118) were shown to be 5D specific (Figure 2). 5DL specific pre-miRNA (miR169, miR5085) coding sequences were shown to be located between the centromer and the breakpoint present in 5DL-7 (FL : 0.29) deletion line (Figure 3). Comparative quantification of gene copy number of pre-miR5070 in CS and nullitetrasomic lines revealed approximately %9 of the miR5070 coding regions were located on 5D chromosome (Figure 4). 5D-specific pre-miR2118 was shown to be expressed from the leaf tissue of CS grown under standard greenhouse conditions (Figure 5). Homologs of this miRNA have been previously shown to be involved in disease resistance and production of secondary siRNAs [66]–[68]. Other miRNAs included in this study (miR169, miR5085, miR6220, miR2118) may also be expressed, under stress conditions, in other wheat tissues and/or at different developmental stages. For instance, in several reports, miR169 was implicated in a broad range of stress responsive mechanisms including nitrogen starvation, arsenic, salt and drought stresses and response to virus infection [69]–[73].

### Conclusion

Here we performed the first systematic identification of miRNAs in *T. aestivum* chromosome 5D through the use of next-generation sequencing data. In this study we found 55 putative miRNAs, of which 14 were not previously identified in wheat. Their potential targets were also predicted, and drought-related miRNA targets were detected. Moreover, *in silico* expression analysis of the predicted miRNAs gave EST hits for 6 out of 55 miRNAs. Furthermore we verified the 5D chromosome localization of 5 miRNAs, 3 of which were found to be 5D specific. Among these, expression of miR2118 was experimentally shown.

The findings from this study will contribute to future research on wheat miRNA function.

## Materials and Methods

### miRNA reference set

A total of 5,940 miRNAs from 67 plant species deposited in the current release of miRBase have been downloaded (http://www.mirbase.org/) and compared to bread wheat (*Triticum aestivum* L.) chromosome 5D survey sequence data in order to find all miRNAs represented in *T.aestivum.*After identical miRNA sequences had been removed,3,228 unique sequences corresponding to 1,556 miRNA families were remained. To date 42 miRNAs were shown to be present in *T.aestivum* [42]–[44].

### Wheat chromosome 5DS & 5DL sequences

Long and short arm of *T.aestivum* (cv Chinese Spring) flow sorted chromosome 5D were sequenced by using GS Titanium Rapid Library Preparation Kit, the GS FLX Titanium LV emPCR Kit and GS FLX Titanium Sequencing (XLR70) Kit following the manufacturer’s instructions(Roche Diagnostics). A total of 3,208,630 reads; 937,264 from 5DS, 2,271,366 for 5DL were obtained representing 1.34x and 1.61x coverage for 5DS and 5DL respectively. All sequence reads were submitted to the EBI Sequence Read Archive, accession number ERP002330 (http://www.ebi.ac.uk/ena/data/view/ERP002330).Two separate databases were generated from 454 GS FLX sequence reads using the BLAST+ stand-alone toolkit, version 2.2.25 [74].

### Computational prediction of conserved miRNAs in wheat chromosome 5D

Conserved miRNA sequences were identified with the strategy described in [3], [48], [75] using two in-house Perl scripts: SUmirFind and SUmirFold (For current versions of both scripts please contact S.Lucas (slucas@sabanciuniv.edu). A total of 3228 mature miRNA queries were blasted separately against the sequence databases generated for 5DS and 5DL. SUmirFind uses blastn algorithm with parameters optimized for short-query sequences (word size:7;dust filter: off; e-value: 1,000). The program also eliminates the hits giving more than 3 mismatches to a published mature miRNA query sequence. SUmirFind results were recorded in table format including miRNAs giving hit to sequence reads. These read sequences were subjected to UNAFnew2, edited version of UNAFold (an implementation of Zuker algorithm [76]) using the other Perl script, SUmirFold. The secondary structures of the hit sequences were first predicted and sequences with more than 6 mismatches to the mature miRNA were removed. The remaining sequences containing the miRNA sequences were re-folded and checked whether they fit the putative miRNA criteria described in [17], [37], [48], [77]. After the manual elimination of multi-branch loops, following characteristics were determined and given in a table format: the new miRNA sequence, conserved miRNA sequence, pre-miRNA sequence, sequence ID, mature miRNA length, pre-miRNA length, number of mismatches to the query, pre-miRNA stem-loop start and end sites, hairpin location, MFE (ΔG kcal/mol), %GC content and MFEI. Maximum, minimum, and average of these values were calculated separately first and later combined for 5DS and 5DL (Data S2: Table 2 and Table 3).

### miRNA representation analysis for both arms in chromosome 5D

The number of sequence reads of 5DS and 5DL which contained potential *T. aestivum* miRNA stem-loop structures were counted and recorded for each miRNA. In order to prevent over-representation, identical hits for the same miRNA were removed. Representation was analyzed both individually and collectively for 5DS and 5DL (Data S3:Table 1).

### Potential miRNA targets

First, all *T. aestivum* predicted miRNAs were searched in miRBase and known targets of homologous miRNAs were listed (Table 1).*T. aestivum* miRNA target prediction was also performed using an online software psRNAtarget containing DFCI Gene Index Release 12. (http://plantgrn.-noble.org/psRNATarget/; [10], [17], [37], [48], [75]) (Data S4: Table 1, 2). Possible target functions of newly identified miRNAs were searched manually using QuickGO (http://www.ebi.ac.uk/QuickGO/), a web based browser for gene ontology terms and annotations which are provided by the UniProt-GOA project at the EBI, and were listed in Table 2.

### Elimination of known repeat sequences encoding miRNAs

In order to screen and mask repetitive elements in all 5DL and 5DS survey sequence reads againsta custom repeat library assembled from the Triticeae Repeat Sequence Database (TREP, release10), RepeatMasker version 3.2.9was used. Sequences matching known repeats were masked and compared with potential *T. aestivum* miRNA sequences to show miRNA representation on repeat regions 5D chromosome arms.

Retroelements and DNA transposons that are present in short and long arms of 5D were listed and compared between each other and potential miRNAreads (Data S5: Table 1, 2 and 3).

### EST analysis for potential miRNAs

For the *in silico* expression of identified miRNAs was analyzed by blasting predicted miRNAs, as queries, against *T. aestivum* EST database in NCBI. All EST matches were compared to the non-redundant protein database at NCBI using blastx in order to find candidate pre-miRNA coding ESTs. Hits with an e-value of less than or equal to 1E-03were considered to be protein-coding, and were eliminated. Predicted miRNA and the accession codes of corresponding EST hits were listed for both arms of chromosome 5D(Table 3).

### Searching for 5D specific expression of miRNAs in *O.sativa* and *B.distachyon*

All *B.distachyon* (International Brachypodium Initiative 2010) and *O.sativa*(International Rice Genome Sequencing Project, last updated in 2010) genomic sequences were downloaded and separate databases were generated for each organism. Predicted 5D miRNAs corresponding to 654 (for 5DL), 428 (for 5DS) unique mature miRNA sequences were blasted against the databases. Predicted miRNAs giving hits to specific chromosomes of *B.distachyon* were listed (Table 4).

### Plant materials and growth conditions

*Triticum aestivum* L. cv. Chinese Spring (AABBDD), its nullitetrasomic and 5D deletion line series were grown in normal greenhouse conditions (16-h light at 22^o^C and 8-h dark at 18^o^C). Seeds were surface sterilized and vernalized in petri dishes for 3–4 days at 4^o^C. Seedlings were transferred to pots containing soil supplemented with 200 ppm N, 100 ppm P and 20 ppm S. Leaf tissue was collected from adult plants (CS, nullitetrasomic and deletion series) and stored at −80^o^C.

Two lines of the nulli-tetrasomic series (N5D-T5A and N5D-T5B : with the genomic constitution of AABBAA and AABBBB, respectively) from Kansas State University were used. These lines lacked homoeologous 5D chromosomes (nullisomic condition) that were replaced by another homoeologous chromosome pair (tetrasomic condition) : 5A and 5B in N5D-T5A and N5D-T5B, respectively.

Four homozygous lines from the group-5 wheat chromosome deletion series (5DS-2, 5DS-5, 5DL-5, 5DL-7) with different deletion breakpoints were also retrieved from the Kansas State University wheat collection and used. The length of the remaining chromosome arm in each deletion line is referred as the 'fraction length' (FL). Corresponding FL values of each deletion line used are given in Figure 3c.

### Plant DNA and RNA material

RNA isolation from frozen CS leaf tissue was carried out using TRI Reagent (Sigma,MO USA) according to the manufacturer’s instructions. Quality and quantity of isolated RNA was measured using a Nanodrop ND-100 spectrophotometer (Nanodrop Technologies, Wilmington, DE, USA). Integrity of the isolated RNA was confirmed by separating the major rRNA bands in agarose gels. DNase treatment of 1 µg of total RNA was performed in 10 µl reaction mixture with 1 U of DNase I dioxyribonuclease I (Fermentas). First strand cDNA was synthesized from 100 ng of DNase treated RNA with RevertAid H- M-MuLV RT (Fermantas).

Genomic DNA isolation from frozen leaf tissue of wheat (CS, nullitetrasomic and deletion series) was performed using Wizard® Genomic DNA Purification Kit (Madison, WI, USA) according to the manufacturer's instructions.

Flow sorted chromosome arms (5DS and 5DL) were obtained from and J. Doležel and colleagues (IEB, Olomouc, Czech Republic; unpublished). All nucleic acid samples were stored at −20°C.

### End point PCR and RT-PCR screening of predicted pre-miRNAs

To experimentally validate 5D chromosome localization of selected pre-miRNAs (miR169, miR5085, miR2118, miR5070, miR6220), PCR screening was carried out using DNA from flow-sorted 5D chromosome arms. To identify 5D chromosome specific miRNAs, screening of gDNA from CS and nullitetrasomic lines (N5D-T5A and N5D-T5B) for these pre-miRNAs was also performed. Additionally, using group-5 deletion series wheat gDNA, 5DL specific pre-miRNAs were screened to determine their location on the chromosome arm.

To check the expression of these pre-miRNAs in adult leaf tissue of wheat plants grown under standard greenhouse conditions, cDNA synthesized from CS RNA was used for RT-PCR.

PCR reactions were performed using 1 ul (10 ng/ul) DNA/cDNA template and were performed in a 20 µl PCR mix including 2 µl 10X Taq buffer (final concentration 1X), 1,6 µl 2.5 mM dNTP (final concentration 0.2 mM), 0,6 µl 10 µM primer (final concentration 300 nM) and 0,1 µl of 5U/µl Taq polymerase (0.5 U). 2.5 mM MgCl_2_ (stock concentration : 25 mM) was used for the amplification of miR6220, miR5070 and miR2118 and this value was optimized to 2 mM and 3 mM for the miR5085 and miR169 amplicons. Thermal cycling setup was adjusted as follows : heated to 95^o^C for 5 minutes; followed by 35 cycles of 95^o^C for 1 minute, 50^o^C/60,5^o^C/62^o^C for 30 seconds and 72^o^C for 30 seconds, followed by 72^o^C for 10 minutes. For amplification of miR2118 and miR5070, the annealing temperature was optimized to 50^o^C and 60,5^o^C, respectively. The annealing temperatures for the remaining miRNAs were optimized to 62^o^C. Primers used for PCR analysis are listed in Data S6 : Table 2.

Separation of PCR products (with 1∶5 ul 6X loading dye) was performed using 3% agarose gels run at 100V.

### Quantitative real time PCR

To quantify pre-miRNA gene copy number and expression in CS, qRT-PCR was performed using FastStart Universal SYBR Green Master (ROX) (Mannheim, Germany) on an Icycler Multicolor Real-time PCR Detection Systems (Bio-Rad Laboratories). For quantification of pre-miR5070, which is located both on 5D and other wheat chromosomes, nullitetrasomic lines were used along with CS to quantify its 5D specific gene copy number. Normalization was performed with BF474284 primers (Forward Primer : CCATACTTGCATCCCCATCT; Reverse Primer : GTGTTGGATGAGCGCATTT), located to the long arm of wheat chromosome 1A.

Using 1ul of DNA/cDNA, quantitative PCR reactions were performed as 20 µL including 10 µL 2× Master mix and 0.6 µL forward/reverse primer mix (300 nM from each). Specified qRT-PCR thermal setup was adjusted as follows: heated to 95°C for 10 min, followed by 40 cycles of 95°C for 15 s, 56/58°C for 30 sec, and 72°C for 30 s, followed by 72°C for 7 min. The annealing temperature was optimized to 56°C for mi6220 and miR2118 quantification. The annealing temperatures for the remaining miRNAs were optimized to 58°C. The melting curves were generated by collecting fluorescence signals from 55°C to 95°C as the temperature increased 0.5°C with a dwell time of 10 seconds for 80 cycles. (Pre-miR2118 gene copy number quantification could not be performed due to the presence of an additional nonspecific band).

For analysis of quantification, PCR efficiency calculations were performed using the program LinRegPCR retrieved from the publication of Rujiter and his colleagues [78].

## Supporting Information

Data S1 Table 1. List of miRNAs that are found in 5DL, 5DS and 4A chromosome. Table 2: List of newly identified miRNAs for both chromosome arms.
(XLSX)

Data S2 Table 1. MFEI table of both chromosome arms for unique pre-miRNA sequences. Table 2: MFEI table of 5D long chromosome arm with representative miRNA sequences. Table 3: MFEI table of 5D short chromosome arm with representative miRNA sequences.
(XLSX)

Data S3 Table 1. Representation of 5D chromosome miRNAs.
(XLSX)

Data S4 Table 1. psRNATarget results for 5DL chromosome arm. Table 2: psRNATarget results for 5DS chromosome arm.
(XLSX)

Data S5 Table 1. Repeat Families of 5D chromosome. Table 2: RepeatMasker program results for 5D long chromosome arm. Table 3: RepeatMasker program results for 5D short chromosome arm.
(XLSX)

Data S6 Table 1. Pre-miRNA sequences selected for experimental validation. Table 2: Primer sequences.
(XLSX)
